# Longitudinal changes and correlates of meeting WHO recommended levels of physical activity in the UK during the COVID-19 pandemic: Findings from the HEBECO study

**DOI:** 10.1371/journal.pone.0273530

**Published:** 2022-08-24

**Authors:** John J. Mitchell, Samuel J. Dicken, Dimitra Kale, Aleksandra Herbec, Emma Beard, Lion Shahab

**Affiliations:** 1 Institute of Epidemiology and Health Care, University College London, London, United Kingdom; 2 Department of Behavioural Science and Health, University College London, London, United Kingdom; 3 SPECTRUM Research Consortium, Usher Institute, Edinburgh, United Kingdom; University of Strathclyde, UNITED KINGDOM

## Abstract

**Background:**

The COVID-19 pandemic has seen repeated government enforced restrictions on movement. This study aimed to evaluate longitudinal trends in physical activity (PA) in a self-selected UK-based sample and identify the key correlates of these trends.

**Methods:**

From 23 April 2020 to 30 January 2021, measures of PA engagement were collected in a sample of 1,947 UK-based adults. Generalised estimating equations (GEE) explored trends in PA engagement over time, and how sociodemographic, health and contextual factors impacted participant’s attainment of World Health Organization (WHO) recommended levels of PA (constituting muscle strengthening activity (MSA), and moderate or vigorous PA (MVPA)).

**Results:**

While one in five achieved the recommended levels of PA in the first UK lockdown in April-June 2020 (19.5%, 95%CI: 17.8–21.3%) and a similar proportion in June-July 2020 (17.7%, 95%CI: 16.1–19.5%), this reduced during the period of eased restrictions in August-September 2020 (15.2%, 95%CI: 13.7–16.9%) and the second UK lockdown in November 2020-January 2021 (14.1%, 95%CI: 12.6–15.9%). Similar trends were observed for MSA and MVPA individually. Better quality of life, higher socioeconomic position and pre-COVID-19 PA levels were associated with meeting the WHO recommended levels of PA, while those living with overweight or obesity, a limiting health condition, or isolating showed the inverse associations. Time-specific associations with MSA or MVPA were observed for gender and age.

**Conclusion:**

Reductions in PA levels throughout the first strict lockdown continued without reversal during the ensuing period. The association of negative change with socioeconomic and health-related indices points towards deepening health inequities during the pandemic.

## Introduction

Most European nations have undergone secondary or tertiary periods of lockdown in early 2021, with state mandated reductions in social contact, outdoor activities, and freedoms in place to reduce the spread of severe acute respiratory syndrome coronavirus 2 (SARS-CoV-2) [1]. One likely consequence of lockdowns has been changes to physical activity (PA) levels. Early evidence from manufacturers of wearable activity-trackers indicates a dose-response effect between the strictness of lockdown measures enacted by regional governments and the severity of reductions in step-count [2, 3]. Since these early findings, an abundance of cross-sectional studies has revealed highly heterogenous changes in PA levels, with some individuals showing improvements in PA levels [4–6] despite an overall downturn in PA [7–16]. This study aimed to corroborate and extend these findings beyond the first strict lockdown and period of eased restrictions in the UK, using an ongoing longitudinal cohort study, the HEalth BEhaviours during the COVID-19 pandemic (HEBECO) study.

Evidence from UK based studies aligns with global trends, showing decreases in individual’s PA across the first lockdown [2, 7, 17–19]. However, early longitudinal studies show mixed evidence of a reversal of this downturn beyond the first strict lockdown [2, 7, 17–19]. Evidence in the UK is not congruous. One study reports a near-universal decrease in PA during lockdown and a near-full recovery in the PA levels of older adults only (≥65) by the period of eased restriction [20]. One further study reports an increase in MVPA, especially in individuals not previously active, and a decrease in walking during lockdown which recovered during the easing of restrictions [21]. These changes likely reflect the variation in measures collected, as well as the short follow-up periods in these studies, in which the acute and varied lifestyle changes from lockdown may have disproportionately persisted depending on location and personal situation.

The health consequences of physical inactivity and sedentary behaviours are serious. The WHO previously named physical inactivity as a leading cause of global disease burden and published recommendations for weekly PA, with a view to reducing this burden [22, 23]. This encompasses engagement in 150 minutes of MVPA weekly (i.e., activities that increase heart rate and make one feel warmer) and two sessions of MSA per week (e.g., strength/resistance training).

Adherence to the guidelines has been shown to reduce cardiovascular disease risk and mortality [24–30]. Increasing PA engagement can support positive change in other health behaviours such as dietary choices, and attaining sufficient sleep [30–36] while preserving mental and physical wellbeing [31, 37]. Indeed, health behaviour changes are often ‘clustered’, e.g. engaging in less exercise is also associated with increased sedentary time and an increase in health risk behaviours such as alcohol consumption and poorer dietary habits [31]. As such, understanding groups at-risk of poorer PA engagement is a necessary step to unveiling the wider health consequences of enforced lockdowns.

Early evidence has also identified correlates of early negative changes in PA engagement. These include being female, coming from a more disadvantaged, or non-white background, having lower educational level, and having poorer physical and mental health [12, 13, 20, 38]. Few studies to date have provided longitudinal evidence beyond the period of eased restrictions, using the framework of WHO recommendations for PA [20, 21, 39] that *i)* corroborates the persistence of associations with these sociodemographic, health and lifestyle factors; *ii)* examines whether poor PA engagement is reversed after lockdown is eased; and *iii)* whether any improvement of PA habits during the inter-lockdown periods is lost during subsequent lockdowns. Lastly, given the new, emerging, unprecedented restructuring of telework and office hours for many worldwide, this question is of global pertinence. This study therefore aimed to address these gaps by answering the questions:

*RQ1*. How has the proportion of individuals attaining WHO recommended levels of total PA, MVPA and MSA, changed since the strict lockdown in April 2020 until January 2021 in a self-selected sample of UK adults?
*RQ2*. Across the period of follow-up, which health, sociodemographic and COVID-19 related situational factors are associated with meeting *i)* WHO recommended levels of total PA, *ii)* WHO recommended levels of MVPA and *iii)* WHO recommended levels of MSA in a self-selected sample of UK adults?

## Materials and methods

### Study design

The HEBECO study is a longitudinal study of health behaviours during the COVID-19 pandemic in the UK. Ethical approval was sought approved by UCL Research Ethics Committee at the UCL Division of Psychology and Language Sciences (PaLS) (CEHP/2020/579) and informed, written consent was provided by all participants prior to enrolment. Recruitment into the baseline wave commenced on 30th April until 14^th^ June 2020. Recruitment was conducted by snowballing via mailing lists at universities and partner organisations. UK-based adults were specifically targeted by advertising on social media platforms and mailing lists for local councils, parliamentary groups related to health, trade unions, sports clubs, charities, and health volunteering groups. The HEBECO cohort has collected data on PA at four different time points, wave 1 in April-May 2020, wave 2 in June-July 2020, wave 3 in August-September 2020 and wave 4 in November 2020-January 2021. Each of these time points has unique situational differences. Wave 1 (April-May 2020; baseline wave) was during the first strict UK lockdown, when non-essential businesses were closed and with a ban on inter-household mixing was in place. Wave 2 (June-July 2020; first follow-up wave) and wave 3 (August-September 2020; second follow-up wave) reflect the return of some individuals to workplaces and some outdoor inter-household mixing in the period of eased restrictions. Wave 4 (November 2020-January 2021; third follow-up wave) was during the second UK lockdown during the early winter and subject to regional differences in restriction severity.

### Participants

The sample included in this study was derived from participants who consented to participate in the HEBECO study. RQ1 only included those participants who contributed the key outcome variables (measures of PA) during the baseline wave (April-June 2020) and wave 3 (August-September 2020), chosen as the two waves of most significance given their starkly different contexts. For RQ2 the sample was further restricted to participants who additionally provided all sociodemographic, health, lifestyle, and COVID-19 situational factors at these same two time points. A final reduced sample of longitudinally complete cases across all waves was used in sensitivity analyses to test for possible bias due to participant attrition.

### Measures

Full details of the measures used are available at https://osf.io/9cmj3/ and within the attached supplementary document.

### Outcome variables

MVPA and MSA engagement was measured using a self-reported questionnaire, adapted from a previously validated tool [40–42] previously used to classify participant PA engagement based on meeting/not meeting WHO guideline levels. Weekly MSA sessions were measured at each time point with the question, ‘*In the past month*, *on average*, *on how many days per week have you performed strength training*?’, Answers were dichotomised into ≥2 sessions vs all others.

Weekly MVPA engagement was derived from weekly session frequency and average session duration. Session frequency was measured using the question, ‘*In the past month*, *on average*, *how many times per week have you done 15 minutes or more of moderate or vigorous aerobic physical exercise*?’, with answers ranging from ‘0’ to ‘14 or more’ in single frequency increments. This upper limit constraint, set to 2 sessions per day on average is in line with previous output from this cohort [11]. Average session duration was measured with the question, ‘*In the past month*, *how long (in minutes) was your average session of moderate or vigorous aerobic physical activity*?’. Answers were recorded on an interval scale ranging from ‘15 minutes’ to ‘480 minutes or more’ in 1- minute increments. The upper limit constraint of 8 hours capped highly atypical responses.

Meeting WHO recommended levels of total PA was a binary composite score of meeting recommended levels of both MVPA and MSA vs all others. Pre-COVID-19 exercise levels were also measured retrospectively at baseline in the same manner. All outcome measures were time-varying. A final count measure of PA sessions was utilised in sensitivity analysis and derived by summing the weekly number of MVPA and MSA sessions reported by participants. This explored whether these associations are broadly consistent when using a linear indication of PA engagement.

### Explanatory factors

Explanatory factors were included based on previous evidence during the first COVID-19 lockdown periods in the UK and Europe [12, 13, 20, 38, 43] and encompassed sociodemographic and lifestyle and health factors. Lastly, exploratory COVID-19 situational factors were also included.

Sociodemographic explanatory variables included age (continuous), gender (female vs male and non-binary) and ethnicity (white vs all others, encompassing; mixed/multiple ethnic groups, Asian/Asian British, Black/African/Caribbean/Black British, Chinese, Arab, Other ethnic group). These were measured at baseline and included as fixed (time-invariant) covariates. Socioeconomic position was also time-invariant, measured at baseline using a novel, composite index based on participants educational level, household income, and housing tenure. A sum score ranging from 0–3 was derived where one point was issued for each of i) attainment of at least A-level or equivalent qualifications, ii) house ownership (outright or mortgage), iii) possessing a total household income of ≥£50,000. An employment status/working from home composite measure was time varying. Those in full or part time employment or education were subdivided into those who can work entirely at home and those who must attend their place of work/study.

Lifestyle and health explanatory variables included Quality of Life which was time-varying and encompassed participant living situation, social relationships, family relationships and psychological wellbeing, each of which was scored on a five point Likert scale ranging from 1, ‘*poor’* to 5, ‘*excellent’*, from which a mean score was derived. Perceived Health Risk from COVID-19 was time varying, operationalised as a dichotomy of ‘major to moderate risk’ and ‘minimal to no risk’. Having a Body mass index (BMI; body mass (*kg*) /height (*m*)^2^) classed as overweight or obese was derived at each time point from baseline height and self-reported body weight at a given wave, dichotomised into living with overweight or obesity (≥25.00kg/m^2^) and all others including ‘don’t know’ and ‘prefer not to say’. A weighted mean of body mass at waves 1 and 3 was used to impute BMI at wave 2 where body mass was not collected. Having a confirmed or suspected COVID-19 infection was time-varying. Presence of a limiting health conditions which might impact on the participant’s ability to engage in PA was measured at baseline and fixed throughout. Lastly, risky health behaviours including high alcohol consumption (>14 units/week) and current smoking were included as time-varying factors.

COVID-19 situational factors included isolating and home-environmental factors which may confound on PA engagement throughout this period. Isolation was measured at all time-points and dichotomised into strict isolation vs all others. Adequate exercise space access was fixed, measured at baseline. Lastly, participants were dichotomised into those living alone or with others at each time point.

### Statistical analysis

Chi-square and *t*-tests were used to compare the participants who fulfilled eligibility criteria (analytic sample) with those in the excluded sample. Analyses were conducted using SPSS 22.0 (IBM, Armonk, NY, USA). For RQ1, time-trend analyses were conducted by fitting generalized estimating equations (GEE) [44] for each outcome (total PA (MVPA and MSA combined) MVPA and MSA respectively) across waves (timepoints), which estimated the proportions of the sample attaining WHO recommended levels of PA without adjustment for covariates. Pairwise comparisons between time points were made using a type III Wald Chi-square test with Šidák correction for family-wise error [44, 45]. Given the binary nature of the outcomes and the repeated measurement of participants, GEE were fitted using the log-link function and using an autoregressive correlation matrix (selected based on study design and best fit, indicated by the Quasilikelihood under the Independence model Criterion (QIC)) [44]. For RQ2, univariate models were first fitted between each factor and outcome variable and included a timepoint*independent variable interaction term to explore time-specific effects. All terms were included in the final model. For significant interactions, a forward selection approach was used whereby only those which improved model fit were included separately in the final model. For nonsignificant independent variables, Bayes Factors (BF) were calculated based on previous evidence exploring the impact of sociodemographic characteristics on PA levels, to differentiate evidence for no-effect from small effects. A cut-off of 1/3 was used for strong evidence of no effect, and 3 for evidence of a small effect [46–49].

Missing data were treated as missing at random. The study protocol was pre-registered on the Open Science Framework (OSF) before analysis (https://osf.io/q2zak/). Deviations from the pre-registered protocol are described in the supplement and are available in the supplement.

Sensitivity analyses using longitudinally complete cases were used to check the robustness of findings. Furthermore, a count variable of PA sessions was used to explore whether associations observed in RQ2 were present with an outcome more indicative of total PA levels to aid in comparison with previous literature.

## Results

### Participant characteristics

The analytic sample of 1,947 UK-based adults, derived from the total of 2,992 HEBECO participants was principally female (70%, N = 1363), middle aged (mean = 50 years, standard deviation (SD) = 14.7 years) and of white ethnicity (95%, N = 1850) (Table 1). Our sample also contained a high proportion (30%, N = 584) of participants in the highest socioeconomic position based on household income, education, and home ownership relative to the proportions that might be expected from ONS census data [50] (S1 File). Some significant differences existed between the analytic and excluded samples. The included sample were significantly younger (*p<0*.*001)*, had more females (*p = 0*.*024)*, fewer smokers (*p<0*.*001)* and more individuals living with overweight or obesity *(p<0*.*008)*. Further comparisons are presented in Table 1.

**Table 1:** Participant demographics at baseline.

	Analytic Sample	Excluded Sample	Comparison
	N = 1,947	N = 1,045	P-value
**Mean Age (SD)**	**50.61 (14.66)**	**42.93 (15.68)**	**<0.001** *
**Female Gender, % (N)**	**70% (1363)**	**66% (690)**	**0.024** *
**White Ethnicity, % (N)**	**95% (1850)**	**91% (951)**	**<0.001** *
**Country of residence, % (N)**			0.36
England	**86% (1674)**	**85% (888)**	
Scotland	**8% (156)**	**7% (73)**	
Wales	**5% (97)**	**6% (63)**	
Northern Ireland	**1% (19)**	**2% (21)**	
**Composite Socioeconomic Index, % (N)**			**<0.001** *
Highest	**30% (584)**	**26% (272)**	
Upper	**31% (604)**	**34% (355)**	
Lower	**26% (506)**	**32% (334)**	
Lowest	**3% (59)**	**8% (84)**	
**Employment Status, % (N)**			**<0.001** *
Employed & Working from Home	**51% (993)**	**52% (543)**	
Employed & Attending Workplace	**18% (350)**	**23% (240)**	
Unemployed, Retired or full-time Carer	**31% (604)**	**25% (261)**	
**Balcony/Garden Access at home % (N)**	**73% (1421)**	**68% (711)**	**0.007** *
**Living Alone % (N)**	**17% (331)**	**16% (167)**	0.36
**Time-varying characteristics at baseline**.			
**Smoker % (N)**	**14% (273)**	**28% (293)**	**<0.001** *
**Alcohol Consumption % (N)**			0.24
Greater than 14 weekly units	**19% (370)**	**20% (209)**	
Less than or equal to 14 weekly units	**81% (1577)**	**80% (836)**	
**Confirmed or Suspected COVID-19 Infection % (N)**	**20% (389)**	**23% (240)**	**0.041** *
**Strict Isolation % (N)**	**7% (136)**	**7% (73)**	0.95
**Composite Quality of Life Index (0–5) Mean (SD)**	**3.43 (0.47)**	**3.24 (0.88)**	**<0.001** *
**BMI (Overweight) % (N)**	**54% (1051)**	**50% (523)**	**0.008** *
**Chronic condition or Disability % (N)**	**11% (214)**	**11% (115)**	0.70
**Meeting WHO Recommended Total PA % (N)**	**20% (389)**	**20% (209)**	0.86
**Meeting WHO Recommended MVPA % (N)**	**44% (857)**	**41% (428)**	0.25
**Meeting WHO Recommended MSA % (N)**	**34% (662)**	**35% (366)**	0.84

*Significant differences in included and excluded sample at ɑ ≤ 0.05 as indicated by *t*-test of means or Pearson Chi-Square test of difference of proportions (χ2).

### Meeting WHO recommended levels of total PA, MVPA and MSA

**Fig 1:**
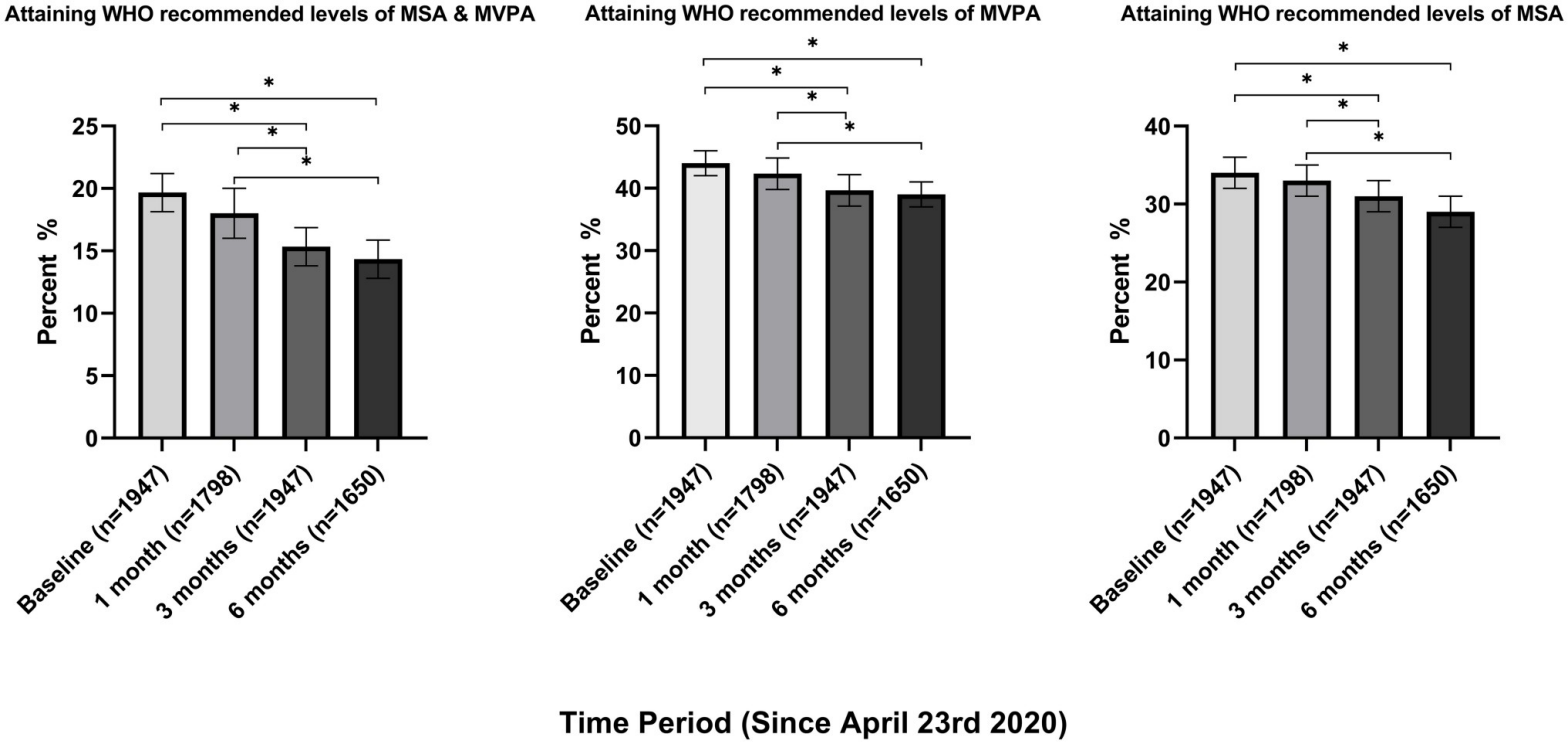
Time trend analysis of PA levels across the COVID-19 pandemic. Time-trends in proportions of participants meeting MVPA, MSA and both WHO PA guidelines & Wald χ2 pairwise comparison with Šidák correction *p < 0.05.

Time-trend analysis demonstrated a steady decline in the proportions of individuals meeting WHO recommended levels of all PA types across the study period (Fig 1). The proportion of individuals attaining WHO recommended levels of total PA decreased from baseline wave 1 (April-May) (19.5%, 95%CI: 17.8–21.3%) to wave 2 (June-July) (17.7%, 95%CI: 16.1–19.5%), wave 3 during the period of eased restriction (Aug-Sept) (15.2%, 95%CI: 13.7–16.9%) and wave 4 during the second UK lockdown (Nov-Jan) (14.1%, 95%CI: 12.6–15.9%). However, no significant differences were observed between the first two or latter two waves. Both MVPA and MSA showed similar decreases across the study period, but again showed no significant differences between waves 1 and 2, or between waves 3 and 4 (Fig 1). This trend persisted in complete case analysis (S1 File).

### Correlates of meeting WHO recommended levels of total PA

**Fig 2:**
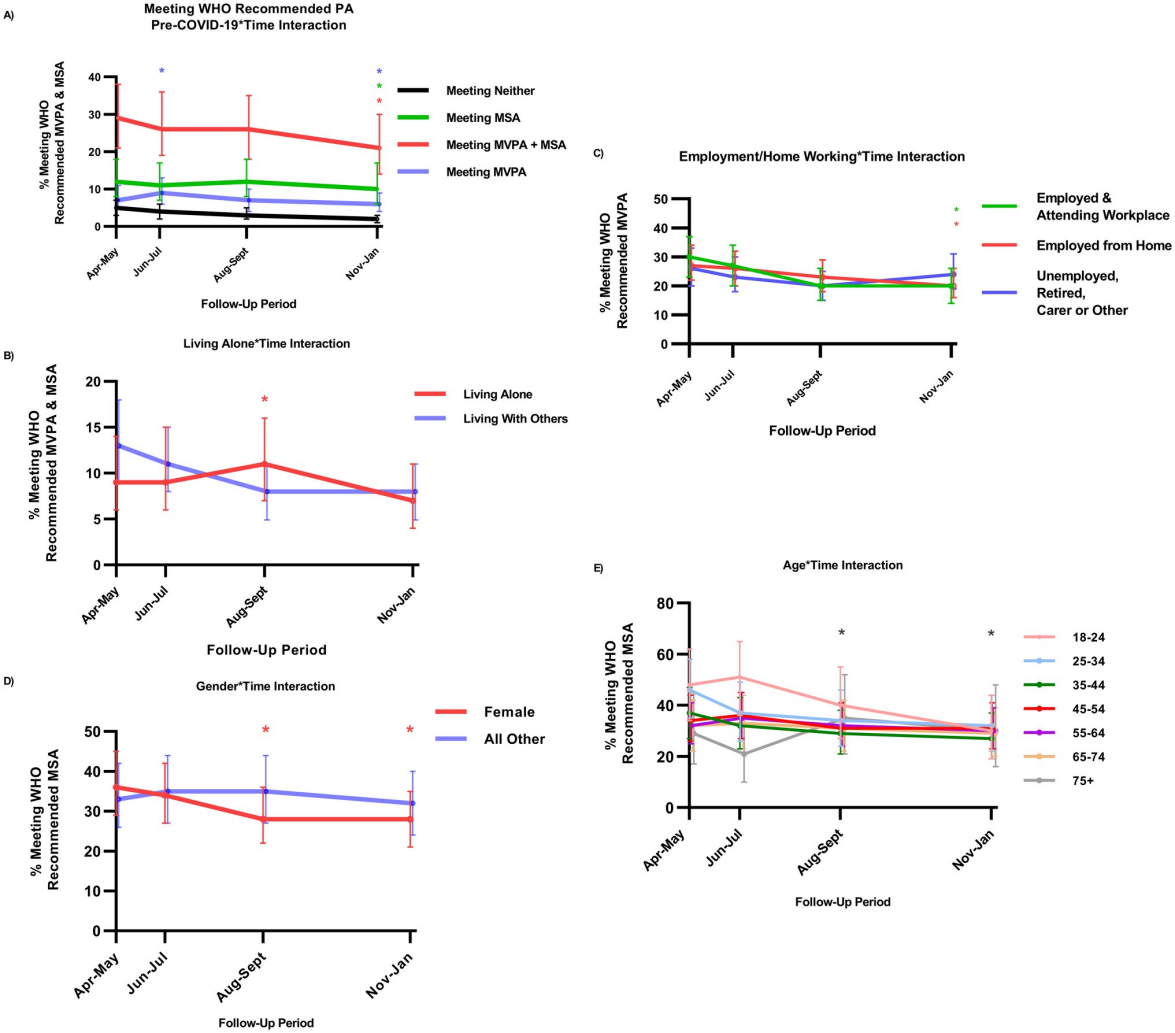
Time-varying associations of factors with meeting total WHO recommended PA levels, MSA & MVPA. A) Meeting any PA recommendation pre-COVID-19 increased odds of meeting WHO recommended levels of PA during the second lockdown. B) Living alone showed higher odds of meeting total WHO recommended levels of PA during the period of eased restriction. C) Working/studying from home or attending a workplace was associated with higher odds of attainment of MVPA during the second lockdown. D) Gender (female) showed lower odds of MSA attainment during the period of eased restriction and second lockdown. E) Age shows a positive association with MSA attainment at these time points * p<0.05.

Sociodemographic and situational factors associated with meeting WHO recommended levels of total PA included socioeconomic position, being in strict isolation and those living alone. The highest two socioeconomic positions were associated with attaining WHO recommended levels of total PA (Table 2). Those in strict isolation showed lower odds of meeting WHO recommended levels of total PA. Living alone showed time-specific effects only, predicting higher total PA attainment specifically during the period of eased restriction in August-September (*p = 0*.*001)* (Fig 2; S1 File). Health and behavioural factors including living with overweight or obesity and a higher perceived risk from COVID-19 infection were negatively associated with attaining total recommended levels of total PA (Table 2). The strongest predictors of meeting WHO recommended levels of total PA during the study period was meeting them prior to the COVID-19 pandemic (Table 2). This relationship with pre-COVID PA levels also showed significant moderation by time, showing stronger associations by the second lockdown (*p = 0*.*037*) and suggesting that failing to meet recommended PA levels pre-pandemic became more strongly associated with not meeting total PA levels as the pandemic progressed (Fig 2; S1 File). Inclusion of time itself in adjusted models confirmed the unadjusted time-trend analysis, as time showed incrementally lower odds of meeting all PA types further into the pandemic (S1 File).

**Table 2:** Factors associated with meeting WHO recommended levels of PA throughout the COVID-19 pandemic.

*Independent Variable*	*Meeting WHO Recommended levels of total PA (both MVPA & MSA)*			*Meeting WHO Recommended levels of MVPA*			*Meeting WHO Recommended levels of MSA*		
*(n = 1908)*	*OR*	*95% CI*	*p-value*	*OR*	*95% CI*	*p-value*	*OR*	*95% CI*	*p-value*
**Sociodemographic Factors**									
**Gender (Female vs all others)**	0.98	(0.79–1.21)	0.85	0.81	(0.69–0.96)	**0.02**	1.13	(0.89–1.44)	0.23
**Age**	1.00	(0.99–1.01)	0.92	1.01	(1.01–1.02)	**<0.001**	0.98	(0.98–0.99)	**0.021**
**Ethnicity (White vs all others)**	0.95	(0.66–1.32)	0.70	1.46	(1.07–1.98)	**0.016**	0.66	(0.45–0.97)	**0.036**
**Employment/Home Working (ref. Unemployed)**			0.80			0.91			0.42
*Employed/Student + working from home*	0.91	(0.70–1.19)	0.47	1.20	(0.88–1.65)	0.55	1.00	(0.79–1.26)	1.00
*Employed/Student + attending workplace*	0.94	(0.69–1.29)	0.14	1.20	(0.88–1.65)	0.25	0.86	(0.66–1.13)	0.28
**Socioeconomic Index (ref. Lowest)**			**0.014**			**<0.001**			0.59
*Highest*	2.32	(1.15–4.69)	**0.019**	1.63	(1.12–2.37)	**0.01**	1.44	(0.77–2.69)	0.25
*Upper*	2.30	(1.14–4.46)	**0.020**	1.43	(1.00–2.05)	0.05	1.39	(0.75–2.58)	0.29
*Middle*	1.69	(0.83–3.46)	0.15	1.05	(0.72–1.52)	0.81	1.29	(0.69–2.40)	0.43
**COVID-19 Situational Factors**									
**Home Exercise Space Access**	0.95	(0.76–1.19)	0.66	0.80	(0.67–0.96)	**0.02**	0.95	(0.78–1.16)	0.64
**Living Alone**	0.66	(0.46–0.96)	0.55	0.93	(0.75–1.16)	0.53	0.84	(0.66–1.07)	0.16
**Isolation (Strict vs all others)**	0.56	(0.38–0.82)	**0.003**	0.41	(0.31–0.56)	**<0.001**	0.98	(0.73–1.31)	0.88
**Risk from COVID-19 (High vs all others)**	0.85	(0.73–0.99)	**0.041**	0.92	(0.81–1.04)	0.19	1.00	(0.88–1.14)	0.99
**Composite Quality of Life Index**	1.30	(1.17–1.44)	**<0.001**	1.27	(1.17–1.38)	**<0.001**	1.29	(1.18–1.41)	**<0.001**
**Lifestyle & Health Factors**									
**BMI (Overweight or obese vs all others)**	0.65	(0.54–0.78)	**<0.001**	0.74	(0.65–0.86)	**<0.001**	0.69	(0.59–0.80)	**<0.001**
**Risk Alcohol Consumption (>14 weekly units)**	0.83	(0.68–1.01)	0.06	0.88	(0.76–1.02)	0.10	0.79	(0.68–0.92)	**0.002**
**Smoking (Smokers vs all others)**	0.74	(0.54–1.01)	0.06	0.90	(0.71–1.11)	0.36	0.77	(0.60–0.99)	**0.039**
**Chronic Health Condition**	0.79	(0.53–1.18)	0.25	0.53	(0.41–0.70)	**<0.001**	0.87	(0.64–1.19)	0.39
**Suspected/Confirmed COVID-19 Infection**	1.00	(0.81–1.22)	0.97	0.98	(0.83–1.15)	0.78	0.98	(0.82–1.16)	0.79
**Meeting WHO Recommended Levels**:			**<0.001**			**<0.001**			**<0.001**
*MSA & MVPA before COVID-19*	8.22	(5.90–11.46)	**<0.001**	4.68	(3.78–5.81)	**<0.001**	7.82	(6.21–9.83)	**<0.001**
*MVPA only before COVID-19*	1.57	(1.11–2.22)	**0.011**	5.24	(4.34–6.33)	**<0.001**	1.08	(0.86–1.36)	0.50
*MSA only before COVID-19*	2.82	(1.95–4.09)	**<0.001**	1.27	(1.01–1.60)	**0.05**	7.27	(5.72–9.25)	**<0.001**
**Time (Ref. Baseline)**			**0.008**			**<0.001**			**0.001**
*1 month*	0.71	(0.54–0.94)	**0.016**	0.87	(0.71–1.08)	0.21	0.69	(0.46–1.05)	0.35
*3 months*	0.42	(0.30–0.59)	**<0.001**	0.70	(0.56–0.87)	**<0.001**	0.61	(0.37–0.99)	0.05
*6 months*	0.28	(0.17–0.46)	**<0.001**	0.91	(0.73–1.15)	0.43	0.81	(0.52–1.26)	**0.003**
Living Alone*Time Interaction (ref. Baseline)			**0.007**						
Pre-COVID PA Levels*Time Interaction Term			**0.037**						
Employment/Home Working*Time Interaction Term						**0.016**			
Age*Time Interaction Term									**0.012**
Gender*Time Interaction Term									**0.018**

Bold values significant at the level ɑ ≤ 0.05 with Type III Wald Chi-square test, see supplementary for full table.

### Correlates of meeting WHO recommended levels of MVPA

Sociodemographic and situational factors including age, gender, white ethnicity, and employment status showed varying associations with meeting WHO recommended levels of MVPA. Employment status showed time-specific associations with attaining sufficient MVPA. Both being employed from home (*p = 0*.*039*) or attending a workplace (*p = 0*.*020*) was associated with less MVPA engagement compared with being unemployed, retired, or a full-time carer during the second UK lockdown in November-January (Fig 2; S1 File). Gender (female) showed a substantial negative association with MVPA attainment while white ethnicity showed a positive association (Table 2). Unusually, having access to adequate exercise space at home was negatively associated with attaining recommended MVPA levels. Again, health and behavioural factors showed significant negative associations with MVPA attainment. Participants living with overweight or obesity showed lower odds of attaining recommended levels of MVPA as did those with a chronic health condition. Pre-COVID-19 PA engagement and higher quality of life again proved the strongest predictors of attaining sufficient MVPA throughout the study period (Table 2).

### Correlates of meeting WHO recommended levels of MSA

Sociodemographic factors including age and being female both showed time-differential, negative effects on MSA at both wave 3 in August-September (*p = 0*.*008; p = 0*.*002*) and wave 4 in November-January (*p = 0*.*001; p = 0*.*027*) (Fig 2; S1 File). Participants of white ethnicity showed substantially reduced odds of attaining recommended levels of MSA, as too did participants living with overweight or obesity (Table 2). Risky health behaviours showed associations specifically with MSA engagement. Participants consuming alcohol above the recommended weekly limits as well as smokers both showed lower odds of meeting recommended levels of MSA (Table 2). Lastly, higher quality of life and attaining sufficient MSA pre-COVID-19 proved strong predictors of meeting recommended MSA levels during the study period.

### Nonsignificant findings

Despite associations between gender and having a chronic health condition and the individual aspects of the PA recommendations, there was no significant findings for an association between these factors and meeting total WHO recommended levels of PA. Bayes Factors were calculated based on previous evidence reporting a negative effect of a chronic health condition (OR = 0.56–0.96) and positive effect of gender (female) (OR = 1.10 to 2.33). BF’s showed there was insufficient evidence to rule out an association between having a chronic health condition and meeting WHO recommended levels of PA (BF = 0.88). Data were also insensitive for gender (BF = 1.06). Age typically appear to show positive associations, compared to which our data consistently provided evidence of no effect (BF = 0.01) [16, 38].

### Sensitivity analysis

To assess for possible bias caused by attrition, final models were completed with complete cases only (S1 File). Trends in PA were robust (S1 File), as were the significance and directions of associations in all models except MSA models, in which alcohol consumption and ethnicity lost significance. To aid comparability of this study with previous literature, and account for the limited sensitivity of the WHO recommendations for total PA engagement, analyses were repeated using a count of weekly PA sessions. Time-trends showed substantive differences at the level of PA sessions (S1 File). Perceived risk from COVID-19, alcohol consumption and smoker status no longer showed significant associations in any model, nor did the time-specific effects of living alone and working from home; however. all other findings remained robust at the level of total PA measured on a linear scale, despite changes to point estimates (S1 File).

## Discussion

Here we provide evidence for changes in the attainment of recommended levels of PA among a self-selected UK-based sample of adults throughout four unique contexts of the COVID-19 pandemic. Proportions of individual’s meeting WHO recommended levels of total PA, MVPA and MSA all showed a decrease throughout April 2020 to January 2021. Previous studies which examined PA habits using minutes of MVPA report recovery for some individuals, specifically older adults during the period of eased restrictions in August-September 2020 [20, 21]. This study identified no significant difference between wave 1 during the first strict lockdown in April-May and wave 2 at the ending of the first lockdown in June-July. However, this study extends beyond these studies, identifying significant drops between these waves and the August-September wave during the period of eased restriction and November-January wave during the second lockdown in the UK [2, 51, 52]. In general, MVPA attainment in this sample was far lower than might be expected from other estimates of PA in the UK population prior to the COVID-19 pandemic, persisting beyond the initial strict lockdown [39, 53, 54]. This may be underpinned by the persistence of changes from the strict lockdown period into daily life beyond the pandemic such as continued home working and the persistence of closures and restrictions on group activities. Crucially, many of the changes to daily life experienced in the first year of the COVID-19 pandemic have persisted to the present, including changes to ordinary working patterns. Given the importance of PA for good health, and the possible implications of even short-term decreases in PA for cardiovascular health [55] these negative trends in PA are therefore in need of continued monitoring in the wake of the pandemic, to ensure PA levels do successfully rebound when sports facilities have fully reopened, especially during warmer seasons.

Many sociodemographic factors were predictive of differential PA engagement. White ethnicity was associated with meeting recommended levels of MVPA, but negatively associated with MSA attainment. Previous studies have observed higher attainment of both MVPA and MSA in white communities [41]. This finding suggests that individuals from different backgrounds may be engaging in PA in different ways during the lockdown.

Despite Bayes Factors suggesting inconclusive evidence for an effect of gender on meeting the WHO guideline levels of total PA, our data does still raise possible gender- differences and socioeconomic inequities in attaining sufficient levels of MVPA and MSA. Females showed lower odds of meeting recommended levels of MVPA, specifically during the period of eased restriction and second lockdown. Increasing age showed differing associations with MVPA and MSA respectively, but Bayes Factors provided evidence for ‘no effect’ of age on meeting WHO recommended PA levels [38]. Evidence is mixed as to the existence, and possible mediators, of gender-differences in PA engagement during the pandemic [16, 56]. Indeed, early cross-sectional data in other UK cohorts indicated significantly higher attainment of MVPA in females [16]; however, subsequent longitudinal studies have suggested little or no difference [57]. One study which performed gender-stratified analysis revealed that females with children and/or those ordinarily receiving support with activities such as childcare, were those who showed lower odds of PA participation [38]. We postulate that greater anxieties regarding accessing outdoor PA without peer support may provide an alternate explanation and avenue of future exploration. Age-related differences have been observed almost unanimously [16, 20] and may be driven by factors such as additional time and freedoms afforded to those who are financially independent which often accompanies older age [58]. However, our study does not corroborate this finding associated with age, despite still identifying strong relationships between greater PA engagement and socioeconomic position and high quality of life [59]. Further to this, participants engaged in negative health behaviours including smoking and excessive alcohol consumption were at greater risk of insufficient PA. These trends therefore align with previous evidence, which suggests the existence of ‘clustering’ of negative health behaviours and an exacerbation of gender-differences and socioeconomic inequities in PA engagement during the COVID-19 pandemic [31].

The most at-risk groups for poor PA engagement included those with a chronic health condition (e.g. stroke, heart disease, dementia), living with overweight or obesity and those with a higher perceived risk from COVID-19, suggesting greater efforts should be made to enable those individuals asked to shield to complete their regular PA, especially given the consequences of even transiently increased sedentarism may be more severe for those with chronic conditions [24, 26, 60–62].

Situational factors were also associated with differing PA engagement. Living alone proved a positive correlate of meeting WHO recommended levels of total PA, specifically during the period of eased restrictions in August-September 2020, perhaps highlighting the importance of social sports and gyms for these individuals to attain their PA. Having adequate space indoors in which to exercise was negatively associated with attaining the recommended levels of MVPA. Perhaps expectedly therefore, MVPA proved the component of participant’s PA requirements most attenuated by being confined to the indoors whilst having to strictly isolate. The strength of association was reduced when looking at total PA sessions, implying that indoor MVPA sessions may be fewer, shorter, or more difficult to attain than MSA, opening the possibility that MSA interventions may prove a useful and more sustainable target for individuals required to shield.

Lastly, pre-COVID-19 PA levels were the most consistent predictors of attainment of recommended levels of all PA types, suggesting relatively strong habits regarding routine PA. Establishing sufficient PA engagement more widely in the wake of the COVID-19 pandemic may therefore remain the optimal target for primary prevention of poor PA attainment during future lockdowns.

### Strengths and limitations

This study provides novel evidence on factors associated with differences in PA engagement across four unique contexts during the pandemic in the UK and holistically explored the factors which may impact PA engagement, using a framework of internationally accepted recommendations for clinically meaningful PA.

Significant limitations, however, do exist. The chosen measures of PA utilised in this study did ensure study brevity and enabled repeated scoring of participants on a widely used benchmark of PA [40]. However The WHO guidelines for PA are binary, thus not reflecting the total daily energy expenditure which is known to be a significant modulator of the beneficial effects of PA [24], and even small positive changes are likely better than no change. For this reason, this study is also not directly comparable with studies examining total daily movement. For instance, sedentary time, which has been shown to have independent, negative health effects [24], was not measured. Further studies should address this with objectively measured PA and sedentary time, while adopting the WHO recommendations as a standard.

The period of eased restriction and subsequent lockdowns beyond the first strict UK-wide lockdown varied by region, and thus individuals may have had differing restrictions during wave 4 depending on their region, as well as differing seasonal and weather trends, which were not modelled in this analysis. The wave also encompasses the Christmas period in which participant’s ordinary patterns of activities are likely to be altered The time points themselves, included in each model was inversely associated with participant PA levels, and likely partially reflects these otherwise uncaptured factored. Several factors were necessarily coded as binary, including alcohol consumption and smoking, which does not account for the significant heterogeneity within these health behaviours. Lastly, use of a self-selected sample, the demographics of which do not accurately represent those of the wider UK population, and the reliance on self-reported measures opens the opportunity for both sampling and social desirability biases.

## Conclusion

This study used PA data contributed by a self-selected UK-based sample to measure population level estimates and correlates of meeting the WHO recommended levels of PA engagement over an eight-month period since April 2020. We observed population-level decreases in PA across the study period, without recovery during the periods outside of lockdown. Several at-risk groups were also identified with consistently lower odds of meeting the WHO recommended levels of PA, which could lead to greater health inequities over time as the UK moves into a new working landscape with majorly restructured telework, office work and home life balance. Future research should make efforts to further discern the situational and motivational reasons behind differences in these at-risk groups to inform future interventions and aid PA promotion in this new landscape.

## Supporting information

S1 File (DOCX)

## References

[1] Nørgaard SK, Vestergaard LS, Nielsen J, Richter L, Schmid D, Bustos N, et al. Real-time monitoring shows substantial excess all-cause mortality during second wave of COVID-19 in Europe, October to December 2020. Euro Surveill. 2021;26(2). Epub 2021/01/16. doi: 10.2807/1560-7917.ES.2021.26.1.2002023 . 33446304 PMC7809719

[2] Pépin JL, Bruno RM, Yang RY, Vercamer V, Jouhaud P, Escourrou P, et al. Wearable Activity Trackers for Monitoring Adherence to Home Confinement During the COVID-19 Pandemic Worldwide: Data Aggregation and Analysis. J Med Internet Res. 2020;22(6):e19787. Epub 2020/06/06. doi: 10.2196/19787 . 32501803 PMC7307323

[3] Tison GH, Avram R, Kuhar P, Abreau S, Marcus GM, Pletcher MJ, et al. Worldwide Effect of COVID-19 on Physical Activity: A Descriptive Study. Ann Intern Med. 2020;173(9):767–70. Epub 2020/07/01. doi: 10.7326/M20-2665 . 32598162 PMC7384265

[4] Constandt B, Thibaut E, De Bosscher V, Scheerder J, Ricour M, Willem A. Exercising in Times of Lockdown: An Analysis of the Impact of COVID-19 on Levels and Patterns of Exercise among Adults in Belgium. Int J Environ Res Public Health. 2020;17(11). Epub 2020/06/14. doi: 10.3390/ijerph17114144 . 32532013 PMC7312512

[5] Ernstsen L, Havnen A. Mental health and sleep disturbances in physically active adults during the COVID-19 lockdown in Norway: does change in physical activity level matter? Sleep Med. 2020. Epub 2020/09/22. doi: 10.1016/j.sleep.2020.08.030 . 32951994

[6] Cancello R, Soranna D, Zambra G, Zambon A, Invitti C. Determinants of the Lifestyle Changes during COVID-19 Pandemic in the Residents of Northern Italy. Int J Environ Res Public Health. 2020;17(17). Epub 2020/09/03. doi: 10.3390/ijerph17176287 . 32872336 PMC7504331

[7] Gallè F, Sabella EA, Ferracuti S, De Giglio O, Caggiano G, Protano C, et al. Sedentary Behaviors and Physical Activity of Italian Undergraduate Students during Lockdown at the Time of CoViD-19 Pandemic. Int J Environ Res Public Health. 2020;17(17). Epub 2020/08/29. doi: 10.3390/ijerph17176171 . 32854414 PMC7504707

[8] Bertrand L, Shaw KA, Ko J, Deprez D, Chilibeck PD, Zello GA. The impact of the coronavirus disease 2019 (COVID-19) pandemic on university students’ dietary intake, physical activity, and sedentary behaviour. medRxiv. 2021:2021.01.12.21249608. doi: 10.1139/apnm-2020-0990 33449864

[9] Deschasaux-Tanguy M, Druesne-Pecollo N, Esseddik Y, de Edelenyi FS, Allès B, Andreeva VA, et al. Diet and physical activity during the COVID-19 lockdown period (March-May 2020): results from the French NutriNet-Santé cohort study. medRxiv. 2020:2020.06.04.20121855. doi: 10.1101/2020.06.04.20121855 PMC7989637 33675635

[10] Górnicka M, Drywień ME, Zielinska MA, Hamułka J. Dietary and Lifestyle Changes During COVID-19 and the Subsequent Lockdowns among Polish Adults: A Cross-Sectional Online Survey PLifeCOVID-19 Study. Nutrients. 2020;12(8). Epub 2020/08/07. doi: 10.3390/nu12082324 . 32756458 PMC7468840

[11] Herbec A, Schneider V, Fisher A, Kale D, Shahab L, Lally P. Correlates of and changes in aerobic physical activity and strength training before and after the onset of COVID-19 pandemic in the UK—findings from the HEBECO study. medRxiv 2021. p. 2021.01.16.21249925. 10.1136/bmjopen-2021-054029 PMC9226469 35732391

[12] Knell G, Robertson MC, Dooley EE, Burford K, Mendez KS. Health Behavior Changes During COVID-19 Pandemic and Subsequent "Stay-at-Home" Orders. Int J Environ Res Public Health. 2020;17(17). Epub 2020/09/03. doi: 10.3390/ijerph17176268 . 32872179 PMC7504386

[13] López-Bueno R, Calatayud J, Casaña J, Casajús JA, Smith L, Tully MA, et al. COVID-19 Confinement and Health Risk Behaviors in Spain. Front Psychol. 2020;11:1426. Epub 2020/06/26. doi: 10.3389/fpsyg.2020.01426 . 32581985 PMC7287152

[14] Luciano F, Cenacchi V, Vegro V, Pavei G. COVID-19 lockdown: physical activity, sedentary behaviour and sleep in Italian medicine students. Eur J Sport Sci. 2020:1–22. Epub 2020/10/29. doi: 10.1080/17461391.2020.1842910 . 33108970

[15] Ong JL, Lau T, Massar SAA, Chong ZT, Ng BKL, Koek D, et al. COVID-19 Related Mobility Reduction: Heterogenous Effects on Sleep and Physical Activity Rhythms. Sleep. 2020. Epub 2020/09/13. doi: 10.1093/sleep/zsaa179 . 32918076 PMC7543649

[16] Smith L, Jacob L, Butler L, Schuch F, Barnett Y, Grabovac I, et al. Prevalence and correlates of physical activity in a sample of UK adults observing social distancing during the COVID-19 pandemic. BMJ Open Sport Exerc Med. 2020;6(1):e000850. Epub 2021/07/01. doi: 10.1136/bmjsem-2020-000850 . 34192006 PMC7358093

[17] Yang Y, Koenigstorfer J. Determinants of physical activity maintenance during the Covid-19 pandemic: a focus on fitness apps. Transl Behav Med. 2020. Epub 2020/09/15. doi: 10.1093/tbm/ibaa086 . 32926160 PMC7797716

[18] Wang X, Lei SM, Le S, Yang Y, Zhang B, Yao W, et al. Bidirectional Influence of the COVID-19 Pandemic Lockdowns on Health Behaviors and Quality of Life among Chinese Adults. Int J Environ Res Public Health. 2020;17(15). Epub 2020/08/05. doi: 10.3390/ijerph17155575 . 32748825 PMC7432516

[19] Castañeda-Babarro A, Arbillaga-Etxarri A, Gutiérrez-Santamaría B, Coca A. Physical Activity Change during COVID-19 Confinement. Int J Environ Res Public Health. 2020;17(18). Epub 2020/09/25. doi: 10.3390/ijerph17186878 . 32967091 PMC7558959

[20] McCarthy H, Potts HWW, Fisher A. Physical Activity Behavior Before, During, and After COVID-19 Restrictions: Longitudinal Smartphone-Tracking Study of Adults in the United Kingdom. J Med Internet Res. 2021;23(2):e23701. Epub 2020/12/22. doi: 10.2196/23701 . 33347421 PMC7861037

[21] Janssen X, Fleming L, Kirk A, Rollins L, Young D, Grealy M, et al. Changes in physical activity, sitting and sleep across the COVID-19 national lockdown period in Scotland. medRxiv. 2020:2020.11.05.20226381. doi: 10.3390/ijerph17249362 33327556 PMC7765067

[22] Bull FC, Al-Ansari SS, Biddle S, Borodulin K, Buman MP, Cardon G, et al. World Health Organization 2020 guidelines on physical activity and sedentary behaviour. British journal of sports medicine. 2020;54(24):1451–62. doi: 10.1136/bjsports-2020-102955 33239350 PMC7719906

[23] World Health O. WHO guidelines on physical activity and sedentary behaviour. Geneva: World Health Organization; 2020 2020. 33369898

[24] Young DR, Hivert MF, Alhassan S, Camhi SM, Ferguson JF, Katzmarzyk PT, et al. Sedentary Behavior and Cardiovascular Morbidity and Mortality: A Science Advisory From the American Heart Association. Circulation. 2016;134(13):e262–79. Epub 2016/08/17. doi: 10.1161/CIR.0000000000000440 . 27528691

[25] Bennie JA, Shakespear-Druery J, De Cocker K. Muscle-strengthening Exercise Epidemiology: a New Frontier in Chronic Disease Prevention. Sports Med Open. 2020;6(1):40. Epub 2020/08/28. doi: 10.1186/s40798-020-00271-w . 32844333 PMC7447706

[26] Kamada M, Shiroma EJ, Buring JE, Miyachi M, Lee IM. Strength Training and All-Cause, Cardiovascular Disease, and Cancer Mortality in Older Women: A Cohort Study. J Am Heart Assoc. 2017;6(11). Epub 2017/11/02. doi: 10.1161/JAHA.117.007677 . 29089346 PMC5721806

[27] Stamatakis E, Lee IM, Bennie J, Freeston J, Hamer M, O’Donovan G, et al. Does Strength-Promoting Exercise Confer Unique Health Benefits? A Pooled Analysis of Data on 11 Population Cohorts With All-Cause, Cancer, and Cardiovascular Mortality Endpoints. Am J Epidemiol. 2018;187(5):1102–12. Epub 2017/11/04. doi: 10.1093/aje/kwx345 . 29099919

[28] Zhao G, Li C, Ford ES, Fulton JE, Carlson SA, Okoro CA, et al. Leisure-time aerobic physical activity, muscle-strengthening activity and mortality risks among US adults: the NHANES linked mortality study. Br J Sports Med. 2014;48(3):244–9. Epub 2013/10/08. doi: 10.1136/bjsports-2013-092731 . 24096895 PMC10938340

[29] Zhao M, Veeranki SP, Magnussen CG, Xi B. Recommended physical activity and all cause and cause specific mortality in US adults: prospective cohort study. Bmj. 2020;370:m2031. Epub 2020/07/03. www.icmje.org/coi_disclosure.pdf and declare: support from the Innovation Team of “Climbing” Program of Shandong University, Youth Team of Humanistic and Social Science of Shandong University, and National Heart Foundation of Australia future leader fellowship for the submitted work; no financial relationships with any organisations that might have an interest in the submitted work in the previous three years; no other relationships or activities that could appear to have influenced the submitted work. 32611588 10.1136/bmj.m2031 PMC7328465

[30] Wu XY, Han LH, Zhang JH, Luo S, Hu JW, Sun K. The influence of physical activity, sedentary behavior on health-related quality of life among the general population of children and adolescents: A systematic review. PLoS One. 2017;12(11):e0187668. Epub 2017/11/10. doi: 10.1371/journal.pone.0187668 . 29121640 PMC5679623

[31] Warburton DER, Bredin SSD. Health benefits of physical activity: a systematic review of current systematic reviews. Curr Opin Cardiol. 2017;32(5):541–56. Epub 2017/07/15. doi: 10.1097/HCO.0000000000000437 . 28708630

[32] Hallgren M, Andersson V, Ekblom Ö, Andréasson S. Physical activity as treatment for alcohol use disorders (FitForChange): study protocol for a randomized controlled trial. Trials. 2018;19(1):106. Epub 2018/02/16. doi: 10.1186/s13063-017-2435-0 . 29444712 PMC5813364

[33] Cornier MA, Melanson EL, Salzberg AK, Bechtell JL, Tregellas JR. The effects of exercise on the neuronal response to food cues. Physiol Behav. 2012;105(4):1028–34. Epub 2011/12/14. doi: 10.1016/j.physbeh.2011.11.023 . 22155218 PMC3260391

[34] Evero N, Hackett LC, Clark RD, Phelan S, Hagobian TA. Aerobic exercise reduces neuronal responses in food reward brain regions. J Appl Physiol (1985). 2012;112(9):1612–9. Epub 2012/03/03. doi: 10.1152/japplphysiol.01365.2011 . 22383502

[35] Vanderlinden J, Boen F, van Uffelen JGZ. Effects of physical activity programs on sleep outcomes in older adults: a systematic review. Int J Behav Nutr Phys Act. 2020;17(1):11. Epub 2020/02/07. doi: 10.1186/s12966-020-0913-3 . 32024532 PMC7003368

[36] Rubio-Arias J, Marín-Cascales E, Ramos-Campo DJ, Hernandez AV, Pérez-López FR. Effect of exercise on sleep quality and insomnia in middle-aged women: A systematic review and meta-analysis of randomized controlled trials. Maturitas. 2017;100:49–56. Epub 2017/05/26. doi: 10.1016/j.maturitas.2017.04.003 . 28539176

[37] Guddal MH, Stensland S, Småstuen MC, Johnsen MB, Zwart JA, Storheim K. Physical activity and sport participation among adolescents: associations with mental health in different age groups. Results from the Young-HUNT study: a cross-sectional survey. BMJ Open. 2019;9(9):e028555. Epub 2019/09/07. doi: 10.1136/bmjopen-2018-028555 . 31488476 PMC6731817

[38] Maertl T, De Bock F, Huebl L, Oberhauser C, Coenen M, Jung-Sievers C, et al. Physical Activity during COVID-19 in German Adults: Analyses in the COVID-19 Snapshot Monitoring Study (COSMO). Int J Environ Res Public Health. 2021;18(2). Epub 2021/01/14. doi: 10.3390/ijerph18020507 . 33435497 PMC7827974

[39] Murphy J, McGrane B, Sweeney MR, editors. Physical Activity, Mental Health and Wellbeing of Irish Adolescents during Covid-19 Restrictions. A Re-Issue of the Physical Activity and Wellbeing Study (PAWS)2021.

[40] Yore MM, Ham SA, Ainsworth BE, Kruger J, Reis JP, Kohl HW 3rd, et al. Reliability and validity of the instrument used in BRFSS to assess physical activity. Med Sci Sports Exerc. 2007;39(8):1267–74. doi: 10.1249/mss.0b013e3180618bbe . 17762359

[41] Bennie JA, De Cocker K, Teychenne MJ, Brown WJ, Biddle SJH. The epidemiology of aerobic physical activity and muscle-strengthening activity guideline adherence among 383,928 U.S. adults. Int J Behav Nutr Phys Act. 2019;16(1):34. Epub 2019/04/20. doi: 10.1186/s12966-019-0797-2 . 30999896 PMC6472085

[42] Wen CP, Wai JP, Tsai MK, Yang YC, Cheng TY, Lee MC, et al. Minimum amount of physical activity for reduced mortality and extended life expectancy: a prospective cohort study. Lancet. 2011;378(9798):1244–53. Epub doi: 10.1016/S0140-6736(11)60749-6 . 21846575

[43] López-Bueno R, Calatayud J, Ezzatvar Y, Casajús JA, Smith L, Andersen LL, et al. Association Between Current Physical Activity and Current Perceived Anxiety and Mood in the Initial Phase of COVID-19 Confinement. Front Psychiatry. 2020;11:729. Epub 2020/08/15. doi: 10.3389/fpsyt.2020.00729 . 32793013 PMC7390883

[44] Zeger SL, Liang KY. Longitudinal data analysis for discrete and continuous outcomes. Biometrics. 1986;42(1):121–30. Epub 1986/03/01. . 3719049

[45] Hubbard AE, Ahern J, Fleischer NL, Van der Laan M, Lippman SA, Jewell N, et al. To GEE or not to GEE: comparing population average and mixed models for estimating the associations between neighborhood risk factors and health. Epidemiology. 2010;21(4):467–74. Epub 2010/03/12. doi: 10.1097/EDE.0b013e3181caeb90 . 20220526

[46] Lakens. Improving Inferences about Null Effects with Bayes Factors and Equivalence Tests. 10.1093/geronb/gby065 29878211

[47] van de Schoot R, Kaplan D, Denissen J, Asendorpf JB, Neyer FJ, van Aken MAG. A gentle introduction to bayesian analysis: applications to developmental research. Child Dev. 2014;85(3):842–60. Epub 2013/10/15. doi: 10.1111/cdev.12169 . 24116396 PMC4158865

[48] Stefan AM, Gronau QF, Schönbrodt FD, Wagenmakers EJ. A tutorial on Bayes Factor Design Analysis using an informed prior. Behav Res Methods. 2019;51(3):1042–58. Epub 2019/02/06. doi: 10.3758/s13428-018-01189-8 . 30719688 PMC6538819

[49] Birch HT. Bayes Factor Calculator 2021 [cited 2021 26/03/2021]. https://harry-tattan-birch.shinyapps.io/bayes-factor-calculator/.

[50] (2019) OfNS. Overview of the UK population: August 2019. 2020.

[51] Richardson DL, Duncan MJ, Clarke ND, Myers TD, Tallis J. The influence of COVID-19 measures in the United Kingdom on physical activity levels, perceived physical function and mood in older adults: A survey-based observational study. J Sports Sci. 2020:1–13. Epub 2020/11/27. doi: 10.1080/02640414.2020.1850984 . 33241966

[52] Schuch FB, Bulzing RA, Meyer J, López-Sánchez GF, Grabovac I, Willeit P, et al. Moderate to vigorous physical activity and sedentary behavior change in self-isolating adults during the COVID-19 pandemic in Brazil: A cross-sectional survey exploring correlates. medRxiv. 2020:2020.07.15.20154559. doi: 10.1101/2020.07.15.20154559 PMC8179086 34108999

[53] HSCIC HaSCIC. Health Survey for England 2016 Physical activity in adults. 2017 13 December 2017. Report No.

[54] HSCIC HaSCIC. Health Survey for England 2018 Adult’s health-related behaviours. NHS Digital. 2019.

[55] Nosova EV, Yen P, Chong KC, Alley HF, Stock EO, Quinn A, et al. Short-term physical inactivity impairs vascular function. J Surg Res. 2014;190(2):672–82. Epub 2014/03/19. doi: 10.1016/j.jss.2014.02.001 . 24630521 PMC4096607

[56] Nienhuis CP, Lesser IA. The Impact of COVID-19 on Women’s Physical Activity Behavior and Mental Well-Being. Int J Environ Res Public Health. 2020;17(23). Epub 2020/12/10. doi: 10.3390/ijerph17239036 . 33291530 PMC7729617

[57] Naughton F, Ward E, Khondoker M, Belderson P, Marie Minihane A, Dainty J, et al. Health behaviour change during the UK COVID-19 lockdown: Findings from the first wave of the C-19 health behaviour and well-being daily tracker study. Br J Health Psychol. 2021. Epub 2021/01/08. doi: 10.1111/bjhp.12500 . 33410229 PMC9291054

[58] Mulder BC, de Bruin M, Schreurs H, van Ameijden EJ, van Woerkum CM. Stressors and resources mediate the association of socioeconomic position with health behaviours. BMC Public Health. 2011;11:798. Epub 2011/10/14. doi: 10.1186/1471-2458-11-798 . 21991933 PMC3205066

[59] Schultz WM, Kelli HM, Lisko JC, Varghese T, Shen J, Sandesara P, et al. Socioeconomic Status and Cardiovascular Outcomes: Challenges and Interventions. Circulation. 2018;137(20):2166–78. Epub 2018/05/16. doi: 10.1161/CIRCULATIONAHA.117.029652 . 29760227 PMC5958918

[60] Steptoe A, Shankar A, Demakakos P, Wardle J. Social isolation, loneliness, and all-cause mortality in older men and women. Proc Natl Acad Sci U S A. 2013;110(15):5797–801. Epub 2013/03/27. doi: 10.1073/pnas.1219686110 . 23530191 PMC3625264

[61] Mañas A, Del Pozo-Cruz B, García-García FJ, Guadalupe-Grau A, Ara I. Role of objectively measured sedentary behaviour in physical performance, frailty and mortality among older adults: A short systematic review. Eur J Sport Sci. 2017;17(7):940–53. Epub 2017/05/24. doi: 10.1080/17461391.2017.1327983 . 28532299

[62] Rosas Hernández AM, Alarcón T, Menéndez-Colino R, Martín Maestre I, González-Montalvo JI, Otero Puime Á. Factors affecting exercise program adherence in patients with acute hip fracture and impact on one-year survival. Braz J Phys Ther. 2020;24(6):479–87. Epub 2019/08/06. doi: 10.1016/j.bjpt.2019.07.008 . 31378633 PMC7779947

